# Optimal UAV Deployment and Resource Management in UAV Relay Networks

**DOI:** 10.3390/s21206878

**Published:** 2021-10-16

**Authors:** Sang Ik Han, Jaeuk Baek

**Affiliations:** 1 School of Smart IT, Semyung University, 65 Semyeong-ro, Jecheon-si 27136, Korea; 2 Electronics and Telecommunications Research Institute (ETRI), 218 Gajeong-ro, Yuseong-gu, Daejeon 34129, Korea; jubaek@etri.re.kr

**Keywords:** UAV relay networks, UAV positioning, resource management, transmit time allocation

## Abstract

UAV equipped three-dimensional (3D) wireless networks can provide a solution for the requirements of 5G communications, such as enhanced Mobile Broadband (eMBB) and massive Machine Type Communications (mMTC). Especially, the introduction of an unmanned aerial vehicle (UAV) as a relay node can improve the connectivity, extend the terrestrial base station (BS) coverage and enhance the throughput by taking advantage of a strong air-to-ground line of sight (LOS) channel. In this paper, we consider the deployment and resource allocation of UAV relay network (URN) to maximize the throughput of user equipment (UE) within a cell, while guaranteeing a reliable transmission to UE outside the coverage of BS. To this end, we formulate joint UAV deployment and resource allocation problems, whose analytical solutions can be hardly obtained, in general. We propose a fast and practical algorithm to provide the optimal solution for the number of transmit time slots and the UAV relay location in a sequential manner. The transmit power at BS and UAV is determined in advance based on the availability of channel state information (CSI). Simulation results demonstrate that the proposed algorithms can significantly reduce the computational effort and complexity to determine the optimal UAV location and transmit time slots over an exhaustive search.

## 1. Introduction

As one of the diverse emerging applications of unmanned aerial vehicles (UAV), it can be utilized as an aerial base station (BS) or an aerial relay node in three-dimensional (3D) wireless networks to satisfy the service requirement of the fifth generation (5G) communication [1,2,3,4], such as enhanced Mobile Broadband (eMBB) and massive Machine Type Communications (mMTC). Due to their mobility, versatile UAVs can adjust their locations to improve the connectivity among user equipment (UEs). Easy deployment of UAV enables to construct 3D networks efficiently with terrestrial networks, which can extend the service coverage or accommodate a large number of devices. By introducing a strong air-to-ground line of sight (LOS) channel, UAV can improve the capability of networks through diverse applications such as (1) emergency supports where communication services are unavailable [5], (2) Internet of Things (IoT) platforms where UAV can collect data from distributed IoT devices by saving their transmit power [6,7], (3) terrestrial network supports where UAVs can assist terrestrial BS transmission or device-to-device (D2D) transmissions [8].

In UAV networks, UAV positioning and radio resource allocation are key factors to extend cell coverage and to improve network performance. The locations of UAV BSs determine the coverage area and the number of UEs within its service area, whereas the resource allocation affects the overall performance of networks. Likewise for UAVs as relay node, its location and resource management (e.g., power control and transmit time allocation) are critical to guarantee seamless connectivity to UEs outside BS service areas without performance degradation.

Many studies on UAV BS scenario (UBS) [9,10,11,12,13] have focused on finding the optimal 3D UAV location to take advantage of strong air-to-ground LOS channels. The study [9] uses a circle packing theory to determine optimal locations of UAVs, and maximizes energy efficiency of UAVs. Another study [10], adopts an optimal transfer theory to minimize total transmit power at UAVs, and investigates the effect of UAV height on power efficiency. The authors of [11] propose a spiral algorithm that sequentially determines the locations of multiple UAVs, and their UAV deployment algorithm is shown to outperform other heuristic schemes in terms of performance and computational time. Study [12] analyzes the effect of interference between UAVs and derives the optimal height of UAV that can maximize the coverage of UAVs. The authors of [13] assume a disaster scenario and propose UAV deployment considering the coexistence of aerial and terrestrial BS. Also, recent works [6,14,15] address more complicated problems of optimizing both UAV deployment and resource allocation to improve network performance. In [6], optimal locations of UAVs, cell association and power controls are provided to maximize energy efficiency in IoT communications. The study [14] optimizes both UAV locations and cell association to minimize network delay. Study [15] achieves capacity enhancement in heterogeneous networks by optimizing UAV deployment, load balancing and traffic offload.

Compared to studies on UBS, research on UAV relay network (URN) is in its infancy, and more efforts are required to optimize both UAV deployment and resource allocation for reliable transmission. Especially, the transmit period of each relay transmission link is one of the most crucial factors on URN because it affects both optimal UAV location and the network performance. Studies [16,17] analyze the performance of URN during two transmit time slots and find the optimal height of UAV [16] and UAV operation range numerically [17] to guarantee a reliable relay transmission. However, a relay transmission during two transmit time slots is sometimes insufficient when UE stays far from BS or requires a high level of quality of service (QoS). To deal with this, Ref. [18] adopts multiple transmit time slots (>2) and derives the maximum distance between UAVs to achieve a reliable relay transmission. However, it does not consider the optimal height of UAV and the performance analysis may not be applicable in all circumstances due to a fixed height of UAVs.

Research on URN to optimize both UAV deployment and resource allocation during multiple transmit time slots (>2) can be rarely found due to the following two main reasons; a relay transmission under time-varying channels and a difficulty on joint optimization of UAV deployment and resource allocation. It is impractical to optimize UAV deployment and resource allocation reflecting channel variations within a single time slot. So, joint optimization of UAV deployment and resource allocation for multiple transmit time slots is required even though they depend on each other.

In this paper, we consider with no constraint on the number of overall transmit time slots in URN to joint optimization for UAV deployment and resource allocation. Especially, the throughput of UE within a cell is maximized while guaranteeing a reliable relay transmission to UE in its extended service area. Multiple transmit time slots are utilized in URN, but the minimum number of overall transmit time slots is considered in a relay transmission for efficient resource management and without performance degradation of UEs within its original coverage due to reduced service opportunity by the BS. The formulated joint UAV deployment and transmit time allocation problem is a mixed-integer nonlinear problem, which is difficult to solve and requires huge computational effort to achieve global optimality. To tackle this, a time-varying channel condition is approximated to the channel expectation in URN. The joint optimization problem is decomposed in a sequential manner. As a solution, we propose the fast and practical UAV deployment and transmit time allocation (UDTA) algorithm, which consists of a novel time slot determination (TSD) algorithm and UAV deployment (UD) algorithm that determines the optimal number of transmit time slots and optimal UAV location, respectively. Transmit power at BS and UAV is determined based on channel state information (CSI). To the best of our knowledge, no such work on URN to optimize UAV deployment and resource allocation for generalized multiple transmit time slots is conducted.

The paper is organized as follows. Section 2 describes a URN system model. In Section 3, the joint UAV deployment and transmit time allocation problem for throughput maximization of UEs is formulated. Section 4 optimizes the UAV location for given transmit time slots, and the optimal number of transmit time slots is determined in Section 5. Computational complexity of the proposed algorithm is analyzed in Section 6. Simulation results in Section 7 demonstrate the optimality and low complexity of the proposed algorithm, followed by the conclusion in Section 8.

## 2. System Model

**Figure 1:**
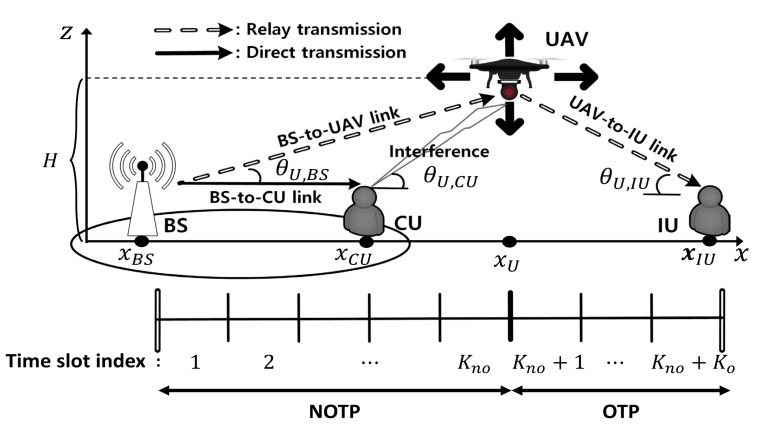
UAV relay network.

We consider a downlink URN, where UAV is used as an aerial relay node to assist BS transmission in the networks, as shown in Figure 1. Two UEs are considered in URN; a UE at the cell edge, denoted as CU, and an isolated UE, denoted as IU. CU can receive a signal from BS through BS-to-CU link, whereas IU can only receive a signal by the relay transmission through BS-to-UAV-to-IU link due to severe pathloss attenuation or blockage between BS and IU. We assume that UAV operates in a half-duplex mode, and hence two transmission phases are considered. UAV receives data from BS in the first transmission phase, and forwards it to IU in the second transmission phase. Multiple time slots are allocated to each transmission phase to guarantee a reliable signal reception at both UAV and IU. Full channel state information (CSI) is assumed at BS, but not at UAV.

We assume that UAV is located at the height of *H* over the line between BS and IU to avoid unnecessary signal attenuation in relay transmissions. In addition, to investigate the effect of interference from UAV to the cell (especially, the worst case of maximum interference to the cell), CU is assumed to be located on the same line between BS and IU for analytical simplicity. Thereby, UAV and ground nodes can be projected onto a plane (i.e., *x*-*z* plane), which reduces to the line between BS and IU (i.e., *x*-axis). The location of ground node *v* (v∈{BS,IU,CU}) can be represented by its *x*-coordinate $x_{v}$, and the UAV location, denoted as U, can be expressed as $U=\left\{x_{U},H\right\}$, where $x_{U}$ is the *x*-coordinate of UAV. $x_{CU}\leq x_{U}\leq x_{IU}$ is assumed to set a strong UAV-to-IU link. Note that the projected two-dimensional (2D) space includes the information on UAV height, so it can clearly reflect the air-to-ground LOS channel characteristics in URN.

### 2.1. Channel Modeling and Assumption

Conventional relay systems where all nodes are located on the ground consider only a ground-to-ground link to characterize channels between nodes, whereas URN consists of not only ground nodes (i.e., BS, CU and IU), but also an aerial node (i.e., UAV). Therefore, an air-to-ground link should be considered along with a ground-to-ground link to characterize channels in URN.

For a ground-to-ground link, a small-scale fading with a pathloss dependent large-scale fading can be used to reflect a rich-scattering environment and a signal attenuation [19]. In URN, the channel between BS and CU is modeled as $h_{BS,CU}d_{BS,CU}^{-\beta_{G}}$, where $h_{BS,CU}∼exp\left(1\right)$ denotes the small-scale fading modeled by Rayleigh distribution, and $d_{BS,CU}^{-\beta_{G}}$ denotes the pathloss dependent large-scale fading. $d_{BS,CU}$ is the distance between BS and CU, and $\beta_{G}$ denotes a pathloss exponent in a ground-to-ground link.

For an air-to-ground link, strong signals in LOS and Non-LOS (NLOS) links dominate the channel characteristics and reduce the randomness of channel fluctuations. Hence, a small-scale fading can be neglected, and only a pathloss dependent large-scale fading in LOS and NLOS links is considered to model an air-to-ground channel in URN [8]. Ref. [20] derives LOS probability of an air-to-ground link between UAV *U* and ground node *v* as

(1) $$\begin{array}{c} {\mathbb{P}}_{los}^{v}=F\left(\theta_{U,v}\right)=\frac{1}{1+C\exp(-B\left[\theta_{U,v}-C\right])}, \end{array}$$

where $\theta_{U,v}$ is an elevation angle between UAV *U* and ground node *v*, as shown in Figure 1. *B* and *C* are coefficients that reflect the characteristics of the environment, such as rural, suburban and urban areas. Compared to a LOS link, an NLOS link experiences an additional signal attenuation of ς [dB]. Therefore, an air-to-ground channel between UAV *U* and ground node *v* can be modeled as $d_{v,U}^{-\beta_{A}}\left({\mathbb{P}}_{los}^{v}+\varsigma {\mathbb{P}}_{nlos}^{v}\right)$, where $d_{v,U}$ is the distance between UAV *U* and ground node *v*, $\beta_{A}$ is a pathloss exponent of air-to-ground link, and ${\mathbb{P}}_{los}^{v}$ and ${\mathbb{P}}_{nlos}^{v}$ are the LOS and NLOS probabilities of the link between UAV *U* and ground node *v* with ${\mathbb{P}}_{nlos}^{v}=1-{\mathbb{P}}_{los}^{v}$.

We assume that the channel condition between BS and CU is better than that between BS and UAV (i.e., $h_{BS,CU}d_{BS,CU}^{-\beta_{G}}>d_{BS,U}^{-\beta_{A}}\left({\mathbb{P}}_{los}^{BS}+\varsigma {\mathbb{P}}_{nlos}^{BS}\right)$). The distance between BS and UAV is much longer than that between BS and CU (i.e., $d_{BS,U}\geq d_{BS,CU}$), because UAV should be located close to IU for a reliable UAV-to-IU link. Due to a long distance between BS and UAV, a pathloss attenuation becomes dominant in the channel condition of LOS link between BS and UAV. Hence, the channel condition between BS and UAV gets worse than that between BS and CU [17].

### 2.2. Transmission Schemes in URN

Based on the result of [17] that a non-orthogonal transmission at BS outperforms an orthogonal transmission in URN in terms of overall throughput of UEs in the cell, we adopt the non-orthogonal transmission at BS in the first transmission phase, where BS transmits a superposition-coded signal to CU and UAV simultaneously [21]. On the other hands, in the second transmission phase, the orthogonal transmission is used at BS and UAV, where BS transmits a signal to CU, and UAV forwards the received data from BS in the first transmission phase to IU. In the rest of this paper, the non-orthogonal transmission phase (NOTP) and the orthogonal transmission phase (OTP) are used to represent the first and second transmission phase, respectively.

### 2.3. Power Control Strategy and Overall Transmit Time Slots

A pairwise power control [22] is adopted at BS during entire transmit time slots of URN to guarantee a required QoS in the cell while supporting a relay transmission to IU. In NOTP, BS allocates $P_{BS,CU}=\rho h_{BS,CU}^{-1}d_{BS,CU}^{\beta_{G}}$ transmit power to BS-to-CU link to guarantee a received signal power of ρ at CU, and the remaining transmit power at BS, $P_{BS,U}$ (i.e., $P_{BS,U}=P_{BS}^{\max}-P_{BS,CU}$), is allocated to BS-to-UAV link, where $P_{BS}^{\max}$ is a maximum transmit power at BS. As a full CSI is available at BS, BS can determine $P_{BS,CU}$ depending on the channel condition in a BS-to-CU link, and then $P_{BS,U}$ can be determined. Similarly, in OTP, BS holds a pairwise power control to guarantee the received signal power of ρ at CU. On the other hand, since UAV has no CSI, UAV uses its maximum transmit power, $P_{U}^{\max}$, to provide seamless communication service to IU.

As illustrated in Figure 1, URN consists of overall $n=K_{no}+K_{o}$ time slots, where NOTP is composed of $K_{no}$ time slots with a time index $k_{no}\in K_{\mathrm{no}}=\left\{1,\ldots ,K_{no}\right\}$ and OTP has $K_{o}$ time slots with a time index $k_{o}\in K_{o}=\left\{K_{no}+1,\ldots ,K_{no}+K_{o}\right\}$. $\left(K_{no},K_{o}\right)$ denotes a pair of time slots for each transmission phase.

## 3. URN: UAV Relay Network

### 3.1. Throughput of CU and IU

At a time slot $k_{no}$ ($\forall k_{no}\in K_{\mathrm{no}}$) in NOTP, BS transmits a superposition-coded signal to CU and UAV simultaneously with transmit power $P_{BS,CU}\left(k_{no}\right)=\rho h_{BS,CU}^{-1}\left(k_{no}\right)d_{BS,CU}^{\beta_{G}}$ and $P_{BS,U}\left(k_{no}\right)=P_{BS}^{\max}-P_{BS,CU}\left(k_{no}\right)$, as explained in Section 2.2. CU can perform the successive interference cancellation (SIC) [21] to eliminate an interference from BS-to-UAV link due to the channel assumption in Section 2.1 (i.e., $h_{BS,CU}\left(k_{no}\right)d_{BS,CU}^{-\beta_{G}}\geq d_{U,BS}^{-\beta_{A}}\left({\mathbb{P}}_{los}^{BS}+\varsigma {\mathbb{P}}_{nlos}^{BS}\right)$). On the other hand, UAV cannot eliminate an interference from BS-to-CU link. Hence, the corresponding signal to interference plus noise ratios (SINRs) at CU, $\psi_{CU}^{no}\left(k_{no}\right)$, and UAV, $\psi_{U}\left(k_{no}\right)$, at a time slot $k_{no}$ in NOTP can be expressed as

(2) $$\begin{array}{cc} \;\;\psi_{CU}^{no}\left(k_{no}\right) & =\frac{\rho }{\sigma_{CU}^{2}}, \end{array}$$

(3) $$\begin{array}{cc} \;\;\psi_{U}\left(k_{no}\right)\; & =\;\frac{P_{BS,U}\left(k_{no}\right)d_{BS,U}^{-\beta_{A}}\left({\mathbb{P}}_{los}^{BS}\left(1-\varsigma \right)+\varsigma \right)}{P_{BS,CU}\left(k_{no}\right)d_{BS,U}^{-\beta_{A}}\left({\mathbb{P}}_{los}^{BS}\left(1-\varsigma \right)\;+\;\varsigma \right)\;+\;\sigma_{U}^{2}}, \end{array}$$

where $d_{BS,U}^{-\beta_{A}}\left({\mathbb{P}}_{los}^{BS}\left(1-\varsigma \right)+\varsigma \right)$ in (3) represents the channel in BS-to-UAV link with LOS probability ${\mathbb{P}}_{los}^{BS}$. $\sigma_{i}^{2}$ indicates the variance of additive white Gaussian noise (AWGN) at a node *i*.

At a time slot $k_{o}$ ($\forall k_{o}\in K_{o}$) in OTP, BS transmits a signal to CU, and UAV relays the received data from BS in NOTP to IU. Therefore, the SINRs at CU, $\psi_{CU}^{o}\left(k_{o}\right)$, and IU, $\psi_{IU}\left(k_{o}\right)$, at a time slot $k_{o}$ in OTP can be given by

(4) $$\begin{array}{cc} \psi_{CU}^{o}\left(k_{o}\right) & =\frac{\rho }{I_{U,CU}+\sigma_{CU}^{2}}, \end{array}$$

(5) $$\begin{array}{cc} \psi_{IU}\left(k_{o}\right) & =\frac{P_{U}^{\max}d_{U,IU}^{-\beta_{A}}\left({\mathbb{P}}_{los}^{IU}\left(1-\varsigma \right)+\varsigma \right)}{\sigma_{IU}^{2}}, \end{array}$$

where $I_{U,CU}≜P_{U}^{\max}d_{U,CU}^{-\beta_{A}}\left({\mathbb{P}}_{los}^{CU}\left(1-\varsigma \right)+\varsigma \right)$ in (4) represents the interference from UAV to CU. IU does not receive any interference from BS in OTP due to severe pathloss attenuation in BS-to-IU link. ${\mathbb{P}}_{los}^{CU}$ and ${\mathbb{P}}_{los}^{IU}$ are the LOS probabilities of UAV-to-CU link and UAV-to-IU link, respectively. Note that we assume that the adjacent cell utilizes different frequency bands from that of the cell of interest to avoid the inter-cell interference, and that other interference received at CU is negligible except that from the link between UAV and IU in OTP, which is dominant.

From (2), (4) and (5), we can find that the SINR at CU in both transmission phases and that at IU in OTP are time-invariant (i.e., $\psi_{CU}^{no}\left(k_{no}\right)=\psi_{CU}^{no}$, $\psi_{CU}^{o}\left(k_{o}\right)=\psi_{CU}^{o}$ and $\psi_{IU}\left(k_{o}\right)=\psi_{IU},\forall k_{no},k_{o}$) due to the pairwise power control and channel characteristics of air-to-ground LOS link. However, the SINR at UAV in NOTP (i.e., (3)) is time-varying for each time slot $k_{no}$ because $P_{BS,U}\left(k_{no}\right)$ and $P_{BS,CU}\left(k_{no}\right)$ vary with the channel condition of BS-to-CU link.

Based on the Shannon capacity theorem [19], the amount of received data at CU, $r_{CU}^{\sum }\left(\left|K_{\mathrm{no}}\right|\right)$, and at UAV, $r_{U}^{\sum }\left(\left|K_{\mathrm{no}}\right|\right)$, in NOTP ($\forall k_{no}\in K_{\mathrm{no}}$) can be obtained using (2) and (3) as

(6) $$\begin{array}{cc} r_{CU}^{\sum }\left(\left|K_{\mathrm{no}}\right|\right) & =\sum_{k_{no}=1}^{K_{no}}f(\psi_{CU}^{no})=f\left(\psi_{CU}^{no}\right)K_{no}, \end{array}$$

(7) $$\begin{array}{cc} r_{U}^{\sum }\left(\left|K_{\mathrm{no}}\right|\right) & =\sum_{k_{no}=1}^{K_{no}}f(\psi_{U}\left(k_{no}\right)), \end{array}$$

respectively, where f(x)≜log(1+x). (6) follows that each time slot has a unit length and $\psi_{CU}^{no}$ is a time-invariant.

Similarly, the amount of received data at CU, $r_{CU}^{\sum }\left(\left|K_{o}\right|\right)$, and at IU, $r_{IU}^{\sum }\left(\left|K_{o}\right|\right)$, in OTP ($\forall k_{o}\in K_{o}$) can be obtained using (4) and (5) as

(8) $$\begin{array}{cc} r_{CU}^{\sum }\left(\left|K_{o}\right|\right) & =\sum_{k_{o}=1}^{K_{o}}f(\psi_{CU}^{o})=f\left(\psi_{CU}^{o}\right)K_{o}, \end{array}$$

(9) $$\begin{array}{cc} r_{IU}^{\sum }\left(\left|K_{o}\right|\right) & =\sum_{k_{o}=1}^{K_{o}}f(\psi_{IU})=f\left(\psi_{IU}\right)K_{o}. \end{array}$$

For the overall time slots *n*, the average data rate of CU, $R_{CU}$ [bps/Hz], can be defined by (6) and (8) as

(10) $$\begin{array}{cc} R_{CU} & =\frac{1}{n}\left(r_{CU}^{\sum }\left(\left|K_{\mathrm{no}}\right|\right)+r_{CU}^{\sum }\left(\left|K_{o}\right|\right)\right), \end{array}$$

and the total amount of received data at IU via relay transmission, $D_{IU}$ [bit/Hz], can be obtained by (7) and (9) as

(11) $$\begin{array}{cc} D_{IU} & =\min(r_{U}^{\sum }\left(\left|K_{\mathrm{no}}\right|\right),r_{IU}^{\sum }\left(\left|K_{o}\right|\right)), \end{array}$$

where (11) follows that the amount of transmitted data through a forwarding link (i.e., UAV-to-IU link) cannot exceed that of received data at UAV via backhaul link (i.e., BS-to-UAV link) in a relay transmission.

### 3.2. Problem Formulation: JUDTAP

The throughput maximization of UEs in URN is equivalent to maximizing $R_{CU}$ while delivering the required amount of data to IU, $D_{req}$, during the minimum number of overall time slots *n* with respect to UAV location $U=\left\{x_{U},H\right\}$ and transmit time slots $K=\{K_{no},K_{o}\}$. Hence, the multi-objective optimization problem, denoted as joint UAV deployment and transmit time allocation problem (**JUDTAP**), can be formulated as (12)

(12) $$\begin{array}{cccc} \mathrm{JUDTAP}: & \max_{U,K}\; & \; & \left[R_{CU},\frac{1}{n}\right] \end{array}$$

(12a) $$\begin{array}{cccc} \; & \;\;\;\;s.t. & \; & D_{IU}\geq D_{req} \end{array}$$

(12b) $$\begin{array}{cccc} \; & \; & & n=K_{no}+K_{o} \end{array}$$

(12c) $$\begin{array}{cccc} \; & \; & & K_{no}\geq 1,K_{o}\geq 1 \end{array}$$

(12d) $$\begin{array}{cccc} \; & \; & & k_{no}\in K_{\mathrm{no}},k_{o}\in K_{o} \end{array}$$

(12e) $$\begin{array}{cccc} \; & \; & & x_{CU}<x_{U}<x_{IU},H\geq 0 \end{array}$$

where multi-objective function implies that the overall number of time slots *n* should be minimized before the average data rate of $R_{CU}$ is maximized, as explained in Section 1. (12a) shows the requirement on the amount of received data at IU. (12b)–(12d) represent the constraints on the number of time slots in URN, and (12e) indicates the possible operation range that UAV can be deployed.

The **JUDTAP** is a mixed-integer nonlinear programming [23] and its combinatorial nature makes the bulk of computational load to find a global optimal solution (i.e., $U^{\mathrm{opt}},K^{\mathrm{opt}}$). In addition, mutual-influence between UAV location and transmit periods in both transmission phases makes it more difficult to be solved. For example, to maximize $R_{CU}$, UAV should be located close to IU to reduce interference from UAV to CU (i.e., $I_{U,CU}$ in (4)). However, it may increase $K_{no}$, eventually *n*, to guarantee a data transmission in BS-to-UAV link (i.e., to satisfy (12a)). Therefore, the **JUDTAP** cannot be solved by optimizing U and K independently due to their close relationships.

One approach to solve the **JUDTAP** is updating U and K iteratively. However, these procedures are not practical and cannot guarantee a convergence to global optimal solution. Therefore, in this paper, we propose a fast and practical algorithm that finds $U^{\mathrm{opt}}$ and $K^{\mathrm{opt}}$ in a sequential manner;

- Step 1: Find the optimal pair of ($K_{no},K_{o}$), $K^{\mathrm{opt}}$, that leads to a minimum *n*.
- Step 2: Determine the optimal location of UAV, $U^{\mathrm{opt}}$.

The details on each step will be presented in Section 4 and Section 5.

### 3.3. Analysis on Relay Transmission during Multiple Time Slots

As explained in the previous section, the relay transmission during multiple transmit time slots makes it difficult to analyze the constraint on BS-to-UAV link in (12a) (i.e., $r_{U}^{\sum }\left(\left|K_{\mathrm{no}}\right|\right)\geq D_{req}$). More specifically, a time-varying small-scale fading in BS-to-CU link changes $\psi_{U}\left(k_{no}\right)$ in (3) and $r_{U}^{\sum }\left(\left|K_{\mathrm{no}}\right|\right)$ in (7) for each time slot $k_{no}$, so it is challenging to find optimal U and K that satisfy $r_{U}^{\sum }\left(\left|K_{\mathrm{no}}\right|\right)\geq D_{req}$. To cope with this issue, we introduce the expected channel model in a ground-to-ground link (i.e., BS-to-CU link in URN) because the effect of random fluctuation by small-scale fading during multiple time slots is negligible and it is impractical to adjust the location of UAV for the short period of each time slot. Therefore, $E\left[h_{BS,CU}\right]d_{BS,CU}^{-\beta_{G}}$ is used to model the channel condition of BS-to-CU link, where $E[h_{BS,CU}]$ represents the expectation of small-scale fading $h_{BS,CU}\left(k\right),\forall k\in K_{\mathrm{no}}\cup K_{o}$. The expected channel model affects the pairwise power control at BS, and the SINR at UAV in (3) as follows.

The pairwise power control at BS is simplified to a fixed power control. In NOTP, BS allocates ${\bar{P}}_{BS,CU}=\rho E{\left[h_{BS,CU}\right]}^{-1}d_{BS,CU}^{\beta_{G}}$ and ${\bar{P}}_{BS,U}=P_{BS}^{\max}-{\bar{P}}_{BS,CU}$ to BS-to-CU link and BS-to-UAV link, respectively. Similarly, in OTP, BS allocates ${\bar{P}}_{BS,CU}=\rho E{\left[h_{BS,CU}\right]}^{-1}d_{BS,CU}^{\beta_{G}}$ to BS-to-CU link.

The time-varying SINR at UAV in NOTP (i.e., $\psi_{U}\left(k_{no}\right),\forall k_{no}$ in (3)) can be replaced into a time-invariant ${\bar{\psi }}_{U}$ due to ${\bar{P}}_{BS,CU}$ and ${\bar{P}}_{BS,U}$. The amount of received data at UAV in (7) can be simplified as ${\bar{r}}_{U}^{\sum }\left(\left|K_{\mathrm{no}}\right|\right)$, where ${\bar{r}}_{U}^{\sum }\left(\left|K_{\mathrm{no}}\right|\right)=f\left({\bar{\psi }}_{U}\right)K_{no}$.

Therefore, the constraint (12a) can be expressed as

$$\begin{array}{cc} \;\; & D_{IU}\geq D_{req} \end{array}$$

(13) $$\begin{array}{cc} \;\; & \Leftrightarrow \frac{{\bar{P}}_{BS,U}d_{BS,U}^{-\beta_{A}}\left({\mathbb{P}}_{los}^{BS}\left(1-\varsigma \right)+\;\;\varsigma \right)}{{\bar{P}}_{BS,CU}d_{BS,U}^{-\beta_{A}}\left({\mathbb{P}}_{los}^{BS}\left(1-\varsigma \right)+\;\;\varsigma \right)\;+\;\sigma_{U}^{2}}\;\;\geq 2^{\frac{D_{req}}{K_{no}}}-\;\;1, \end{array}$$

(14) $$\begin{array}{cc} \;\; & \mathrm{and}\;\;P_{U}^{\max}\frac{d_{U,IU}^{-\beta_{A}}}{\sigma_{IU}^{2}}\left({\mathbb{P}}_{los}^{IU}\left(1-\varsigma \right)+\varsigma \right)\geq 2^{\frac{D_{req}}{K_{o}}}-1, \end{array}$$

where (13) and (14) are obtained from time-invariant ${\bar{r}}_{U}^{\sum }\left(\left|K_{\mathrm{no}}\right|\right)$ and (9), respectively.

After rearranging above two inequalities, the left terms of (13) and (14) can be expressed with respect to LOS probability, which are given as

(15) $$\begin{array}{cc} F(\theta_{U,BS}) & \geq X_{BS}\left(d_{U,BS},K_{no}\right), \end{array}$$

(16) $$\begin{array}{cc} F(\theta_{U,IU)} & \geq X_{IU}\left(d_{U,IU},K_{o}\right), \end{array}$$

where

(17) $$\begin{array}{cc} \; & X_{BS}\left(d_{U,BS},K_{no}\right)\overset{\Delta }{\;=}\frac{\left(2^{\frac{D_{req}}{K_{no}}}-1\right)\frac{\sigma_{U}^{2}}{d_{U,BS}^{-\beta_{A}}\left(1-\varsigma \right)}}{\left(P_{BS}^{\max}-2^{\frac{D_{req}}{K_{no}}}{\bar{P}}_{BS,CU}\right)}\;\;-\;\;\frac{\varsigma }{1-\varsigma }, \end{array}$$

(18) $$\begin{array}{cc} \; & X_{IU}\left(d_{U,IU},K_{o}\right)\overset{\Delta }{\;=}\frac{\left(2^{\frac{D_{req}}{K_{o}}}-1\right)\frac{\sigma_{IU}^{2}}{d_{U,IU}^{-\beta_{A}}\left(1-\varsigma \right)}}{P_{U}^{\max}}-\frac{\varsigma }{1-\varsigma }. \end{array}$$

Based on (15)–(18), a sequential algorithm for Steps 1 and 2 is derived in the following sections.

## 4. UAV Deployment

In this section, we investigate the effect of UAV location on the network performance, and derive the optimal UAV location, $U^{\mathrm{opt}}$. For this purpose, we assume that the transmit time allocation is given (i.e., $\left(K_{no},K_{o}\right)$) and guarantees the existence of UAV locations that can provide a reliable relay transmission to IU. We utilize distance and elevation angle in *x*-*z* plane to reflect the channel characteristics between UAV and ground node in Section 2.1. $\Theta =\{\theta_{U,v}|v\in \left\{BS,CU,IU\right\}\}$ and $D=\{d_{i,j}|i,j\in \left\{BS,U,IU,CU\right\}\}$ represent sets of elevation angles and distances respectively, and the UAV location $U=\left\{x_{U},h\right\}$ in (12) can be expressed as $U=\left\{d_{U,v},\theta_{U,v}\right\}$.

### 4.1. UAV Deployment Problem

Based on (2) and (4), the maximization of multi-objective function for a given time allocation in **JUDTAP** is equivalent to the minimization of interference from UAV to CU (i.e., minimization of $I_{U,CU}$ in (4)). The constraint (12a) can be replaced by (15) and (16), and the constraints (12b)–(12d) can be omitted because the transmit time allocation is given.

Therefore, for a given time allocation, **JUDTAP** reduces to UAV deployment problem (**UDP**), which can be formulated as (19) with respect to Θ and D.

(19) $$\begin{array}{cccc} \mathrm{UDP}: & \min_{\Theta ,D}\; & \; & d_{U,CU}^{-\beta_{A}}\left(F\left(\theta_{U,CU}\right)\left(1-\varsigma \right)+\varsigma \right) \end{array}$$

(19a) $$\begin{array}{cccc} \; & \;\;\;\;s.t. & \; & F\left(\theta_{U,BS}\right)\geq X_{BS}\left(d_{U,BS}|K_{no}\right), \end{array}$$

(19b) $$\begin{array}{cccc} \; & \; & & F(\theta_{U,IU)}\geq X_{IU}\left(d_{U,IU}|K_{o}\right), \end{array}$$

(19c) $$\begin{array}{cccc} \; & \; & & d_{BS,CU}\leq d_{U,BS}cos\left(\theta_{U,BS}\right)\leq d_{BS,IU}. \end{array}$$

The objective function is given by $I_{U,CU}/P_{U}^{\max}$. (19a) and (19b) are the constraints of BS-to-UAV link and UAV-to-IU link, respectively, and derived from (15)–(18) by replacing $X_{BS}\left(d_{U,BS},K_{no}\right)$ and $X_{IU}\left(d_{U,IU},K_{o}\right)$ with $X_{BS}\left(d_{U,BS}|K_{no}\right)$ and $X_{IU}\left(d_{U,IU}|K_{o}\right)$ due to the assumption on time allocation. (19c) represents the UAV operation range that UAV can be deployed (i.e., (12e) of **JUDTAP**).

The optimal solution of **UDP** (i.e., $\Theta^{\mathrm{opt}},D^{\mathrm{opt}}$) determines the optimal UAV location, $U^{\mathrm{opt}}$, for a given time allocation. However, all the elements of Θ and D should be considered simultaneously to find $U^{\mathrm{opt}}$, so no closed-form solution to UDP exists. Therefore, we propose UAV deployment (UD) algorithm, which updates the UAV location iteratively to reach $U^{\mathrm{opt}}$ based on search areas and directions. In the following section, we define search areas and directions for a given UAV location, and investigate them to update the UAV location toward $U^{\mathrm{opt}}$.

### 4.2. Search Areas and Directions

**Figure 2:**
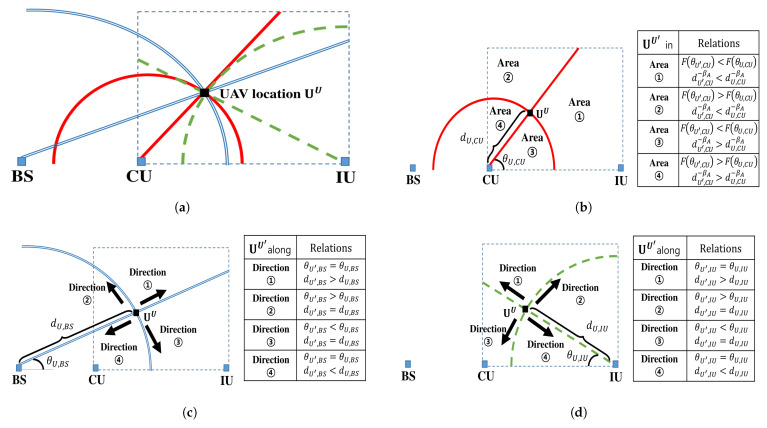
Search areas and directions for updating $U^{U}$ toward $U^{U^{\prime }}$. (**a**) The integrated search areas and directions for $U^{U}$. (**b**) Four search areas for $U^{U^{\prime }}$ based on the objective function of (19) (i.e., link between UAV and CU). (**c**) Four search directions for $U^{U^{\prime }}$ based on the constraint (19a) (i.e., link between BS and UAV). (**d**) Four search directions for $U^{U^{\prime }}$ based on the constraint (19b) (i.e., link between UAV and IU).

For a given location of UAV *U*, we define *search areas* and *directions* using lines and circles as shown in Figure 2, where UAV and ground nodes are placed on the *x*-*z* plane as explained in Section 2. UAVs on a line have the same elevation angle of $\theta_{U,v}$ from ground node *v* (i.e., v∈{CU,BS,IU}), while those on a circle have equal distance of $d_{U,v}$ from ground node *v* to UAV. The line and circle inside dashed-rectangle (i.e., UAV operation range) in Figure 2b define the search areas considering the interference from UAV to CU, while those in Figure 2c,d represent the search directions based on each relay transmission link. All the search areas and directions for a given UAV location are integrated in Figure 2a. The search areas and directions change when a given UAV location is updated. Therefore, we investigate search areas and directions for the given location of UAV *U*, $U^{U}$, to find the updated location of UAV $U^{\prime }$, $U^{U^{\prime }}$.

#### 4.2.1. Search Area

The search areas ➀–➃ in Figure 2b are divided by the line and circle based on CU location and $U^{U}$. UAV *U* should move towards the area where the interference from UAV $U^{\prime }$ to CU (i.e., objective function in (19)) decreases. When UAV *U* moves into Area ➀, both $F(\theta_{U,CU})$ and $d_{U,CU}^{-\beta_{A}}$ in (19) decreases due to smaller $\theta_{U,CU}$ and longer $d_{U,CU}$. Any UAV locations within Area ➀ always reduce the UAV-to-CU interference, hence, Area ➀ is a potential search area for $U^{U^{\prime }}$. On the other hand, all UAV locations in Area ➃ increases both $F(\theta_{U,CU})$ and $d_{U,CU}^{-\beta_{A}}$. Therefore, they cannot decrease the objective function in (19), thereby excluding Area ➃ from potential search areas.

The search areas ➁ and ➂ possess the uncertainty on the interference from UAV $U^{\prime }$ to CU. UAV locations within Area ➁ decrease $d_{U,CU}^{-\beta_{A}}$, but increase the $F(\theta_{U,CU})$, while those within Area ➂ results in the opposite. However, when UAV $U^{\prime }$ is within Area ➁, a pathloss attenuation dominates the LOS connection in BS-to-CU link. More specifically, $F(\theta_{U^{\prime },CU})$ is close to one due to large $\theta_{U^{\prime },CU}$ [20], but $d_{U^{\prime },CU}$ can be sufficiently large so that $d_{U^{\prime },CU}^{-\beta_{A}}$ becomes a dominant factor in the objective function of (19). Therefore, UAV locations within Area ➁ can reduce the UAV-to-CU interference compared to given UAV location $U^{U}$. On the other hand, UAV locations within Area ➂ makes more severe UAV-to-CU interference due to proximity of their locations, hence, Area ➂ cannot be the potential search area.

- Observation 1: The objective function in (19) can be decreased by moving UAV into Area ➀ or ➁ in Figure 2b.

The Observation 1 is directly applicable for a feasible UAV location (which satisfies the constraints (19a) and (19b)) to reduce the UAV-to-CU interference. On the other hands, when UAV location cannot satisfy the constraints (i.e., infeasible UAV location), Observation 1 and the channel condition of relay transmission links should be considered simultaneously to find a feasible UAV location and to reduce the objective function in (19). In the following subsection, we examine the search directions to satisfy the constraints and decrease the objective function in (19) simultaneously.

#### 4.2.2. Search Directions

Although the potential search areas that can be used to find $U^{U^{\prime }}$ from $U^{U}$ are described on Observation 1, there is no clue on $U^{U^{\prime }}$ within the potential area. Hence, the points on a line or a circle within the potential search area are utilized to determine $U^{U^{\prime }}$. In particular, search directions in Figure 2c,d are examined to move UAV *U* into the feasible UAV location $U^{U^{\prime }}$ when $U^{U}$ cannot satisfy the constraints (19a) or (19b).

From the constraints, there are four cases (i.e., $C_{1}$, $\bar{C_{1}}$, $C_{2}$ and $\bar{C_{2}}$) to be considered at $U^{U}$. $C_{1}$ and $C_{2}$ indicate that $U^{U}$ satisfies (19a) and (19b) respectively, while $\bar{C_{1}}$ and $\bar{C_{2}}$ represent that it does not. Each case follows a different search direction in Figure 2c for $C_{1}$ and $\bar{C_{1}}$ and in Figure 2d for $C_{2}$ and $\bar{C_{2}}$.

$C_{1}$ and $C_{2}$ indicate that $U^{U^{\prime }}$ can be found based on Observation 1 to decrease the objective function in (19). In the case of $C_{1}$, Direction ➀ or ➁ in Figure 2c should be selected because they are within the potential search areas ➀ and ➁ in Figure 2b (see Figure 2a). For the same reason, Direction ➀, ➁ or ➃ of Figure 2d should be selected in case of $C_{2}$.

$\bar{C_{1}}$ and $\bar{C_{2}}$ represent that $U^{U}$ cannot satisfy the constraints (19a) and (19b) due to poor channel conditions in BS-to-UAV and UAV-to-IU link, hence resulting in $F\left(\theta_{U,BS}\right)<X_{BS}\left(d_{U,BS}|K_{no}\right)$ and $F\left(\theta_{U,IU}\right)<X_{IU}\left(d_{U,IU}|K_{o}\right)$ respectively. Therefore, to find the feasible UAV location $U^{U^{\prime }}$, $\theta_{U,v}$ should be increased along a circle or $d_{U,v}$ should be decreased along a line in Figure 2c (when v=BS) and Figure 2d (when v=IU). This is because $F(\theta_{U,v})$v∈{BS,IU}, $X_{BS}\left(d_{U,BS}|K_{no}\right)$ and $X_{IU}\left(d_{U,IU}|K_{o}\right)$ are increasing functions of $\theta_{U,v}$, $d_{U,BS}$ and $d_{U,IU}$, respectively.

In the case of $\bar{C_{1}}$, the movement of UAV *U* along Direction ➃ in Figure 2c decreases $d_{U,BS}$, while that along Direction ➁ increases $\theta_{U,BS}$. Direction ➃ always provides the feasible UAV location $U^{U^{\prime }}$ that satisfies (19a), while Direction ➁ could find it only when $X_{BS}\left(d_{U,BS}|K_{no}\right)\leq 1$ because $\max F(\theta_{U^{\prime },BS})=1$. Similarly, in the case of $\bar{C_{2}}$, the movement of UAV *U* along Direction ➁ in Figure 2d increases $\theta_{U,IU}$, while that along Direction ➃ in Figure 2d decreases $d_{U,IU}$. Direction ➁ could find the feasible UAV location $U^{U^{\prime }}$ that satisfies (19b) only when $X_{IU}\left(d_{U,IU}|K_{o}\right)\leq 1$.

- Observation 2 ($C_{1}$): When $U^{U}$ satisfies the constraint (19a), Direction ➀ along a line or Direction ➁ along a circle in Figure 2c should be selected to determine $U^{U^{\prime }}$.
- Observation 3 ($\bar{C_{1}}$): When $U^{U}$ cannot satisfy the constraint (19a), Direction ➃ along a line in Figure 2c always provides the feasible UAV location $U^{U^{\prime }}$, while Direction ➁ along a circle in Figure 2c could find $U^{U^{\prime }}$ only when $X_{BS}\left(d_{U,BS}|K_{no}\right)\leq 1$.
- Observation 4 ($C_{2}$): When $U^{U}$ satisfies the constraint (19b), Direction ➀ or ➃ along a line, or Direction ➁ along a circle in Figure 2d should be selected to determine $U^{U^{\prime }}$.
- Observation 5 ($\bar{C_{2}}$): When $U^{U}$ cannot satisfy the constraint (), Direction ➃ along a line in Figure 2d always provides the feasible UAV location $U^{U^{\prime }}$, while Direction ➁ along a circle in Figure 2d could find $U^{U^{\prime }}$ only when $X_{IU}\left(d_{U,IU}|K_{o}\right)\leq 1$.

#### 4.2.3. Combined Search Directions

The Observations 2–5 should be integrated to consider the constraints (19a) and (19b) together. First, Observations 2 and 4 can be used to decrease the objective function in (19) for the case of $C_{1}$∩$C_{2}$ where $C_{1}$∩$C_{2}$ indicates that $U^{U}$ is a feasible UAV location and satisfies both (19a) and (19b). As in Figure 2, the movement along Direction ➀ in Figure 2c and Direction ➁ in Figure 2d decreases both $F(\theta_{U,CU})$ and $d_{U,CU}^{-\beta_{A}}$ in (19). They have the same properties, but differ on moving along line and circle, respectively. Similarly, the movement along Direction ➁ in Figure 2c and Direction ➀ in Figure 2d decreases $d_{U,CU}^{-\beta_{A}}$ and achieves large $\theta_{U,CU}$, resulting in $F(\theta_{U,CU})\approx 1$. Either direction that has same properties can be selected for the movement towards $U^{\mathrm{opt}}$. However, it is preferable to select search direction moving along a line (i.e., Direction ➀ in Figure 2c and Direction ➀ in Figure 2d) to reduce computation time, which will be discussed in Section 4.3. Note that, UAV locations along Direction ➃ in Figure 2d could break the constraint (19a) due to insufficient height of UAV and small $\theta_{U,BS}$, therefore, it is not an option for $C_{1}$∩$C_{2}$.

- Observation 6 ($C_{1}$∩$C_{2}$): When $U^{U}$ satisfies both the constraints (19a) and (19b), $U^{U^{\prime }}$ will be found along Direction ➀ in Figure 2c or Direction ➀ in Figure 2d.

When $U^{U}$ is infeasible location, there are three cases (i.e., $\bar{C_{1}}$∩$C_{2}$, $C_{1}$∩$\bar{C_{2}}$ and $\bar{C_{1}}$∩$\bar{C_{2}}$) to be considered. However, it is clear that $\bar{C_{1}}$∩$C_{2}$⊂$\bar{C_{1}}$ and $C_{1}$∩$\bar{C_{2}}$⊂$\bar{C_{2}}$, therefore, Observations 3 and 5 will be solutions for each case.

- Observation 7 ($\bar{C_{1}}$∩$C_{2}$): When $U^{U}$ satisfies the constrains (19b), but (19a), Direction ➁ with the condition on $X_{BS}\left(d_{U,BS}|K_{no}\right)$ or Direction ➃ in Figure 2c should be selected to find $U^{U^{\prime }}$ that satisfies (19a).
- Observation 8 ($C_{1}$∩$\bar{C_{2}}$): When $U^{U}$ satisfies the constraints (19a), but (19b), Direction ➁ with the condition on $X_{IU}\left(d_{U,IU}|K_{o}\right)$ or Direction ➃ in Figure 2d should be selected to find $U^{U^{\prime }}$ that satisfies (19b).

Lastly, $\bar{C_{1}}$∩$\bar{C_{2}}$ indicates that $U^{U}$ cannot satisfy both constraints on relay transmission links. Unfortunately, there is no solution based on Observations 3 and 5. For example, if UAV *U* moves along Direction ➃ in Figure 2c to make (19a) satisfied (which is opposite to Direction ➁ in Figure 2d suggested in Observation 5 for the satisfaction of (19b)), it causes $d_{U,IU}<d_{U^{\prime },IU}$ and $\theta_{U,IU}>\theta_{U^{\prime },IU}$, thereby resulting in $F\left(\theta_{U^{\prime },IU}\right)<F\left(\theta_{U,IU}\right)<X_{IU}\left(d_{U,IU}|K_{o}\right)<X_{IU}\left(d_{U^{\prime },IU}|K_{o}\right)$ (i.e., (19b) is still not satisfied). Similarly, other search directions on Observations 3 and 5 also cannot simultaneously improve both relay transmission links, so we declare that no feasible UAV location exists for the case of $\bar{C_{1}}$∩$\bar{C_{2}}$. To deal with this issue, more transmit time slots should be allocated to the relay transmission, which will be discussed in Section 5.

- Observation 9 ($\bar{C_{1}}$∩$\bar{C_{2}}$): When $U^{U}$ cannot satisfy both constraints (19a) and (19b), no feasible UAV location $U^{U^{\prime }}$ exists without allocating more transmit time slots to relay transmission.

### 4.3. UAV Deployment (UD) Algorithm

**Figure 3:**
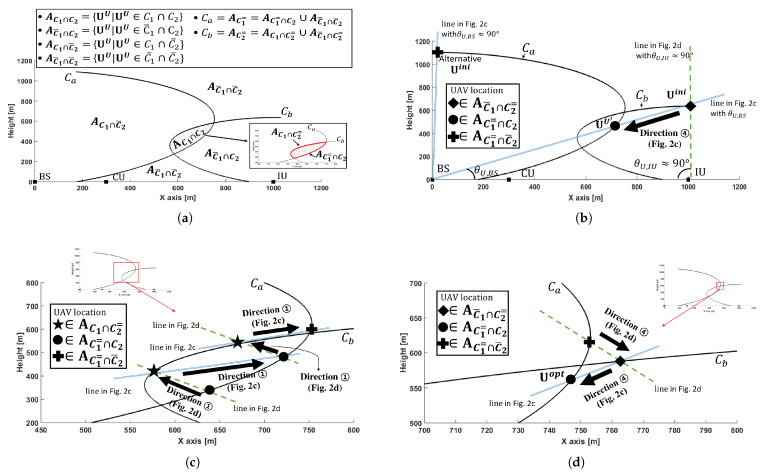
The UAV deployment (UD) algorithm. (**a**) Three areas for $U^{U}$ with respect to constraints (19a) and (19b). $U^{opt}$ is within $A_{C_{1}\cap C_{2}}$ (specifically, either $A_{C_{1}^{=}\cap C_{2}}$ or $A_{C_{1}\cap C_{2}^{=}}$). (**b**) [Step 1] on UD algorithm to determine $U^{U^{\prime }}=U^{f}$ from $U^{U}=U^{ini}$. (**c**) [Step 2] on UD algorithm to update $U^{f}$ until $U^{U^{\prime }}$ is determined outside $A_{C_{1}\cap C_{2}}$. (**d**) [Step 3] on UD algorithm to determine $U^{opt}$ and terminate the algorithm.

In this section, we propose a novel UAV deployment (UD) algorithm for a given time allocation based on search directions. The constraints (19a) and (19b) are described graphically in Figure 3a as parabolic curves $C_{a}$ and $C_{b}$, which are drawn with an equality in (19a) and (19b) respectively. The UAV locations inside $C_{a}$ and $C_{b}$ satisfy the constraints (19a) and (19b) respectively, therefore, areas for $C_{1}\cap C_{2}$, $C_{1}\cap \bar{C_{2}}$, $\bar{C_{1}}\cap C_{2}$, and $\bar{C_{1}}\cap \bar{C_{2}}$ (i.e., $A_{C_{1}\cap C_{2}}$, $A_{C_{1}\cap \bar{C_{2}}}$, $A_{\bar{C_{1}}\cap C_{2}}$, and $A_{\bar{C_{1}}\cap \bar{C_{2}}}$) can be defined as in Figure 3a. In particular, $A_{C_{1}\cap C_{2}}$ (see dashed area in Figure 3a) is of special interest to find the optimal UAV location $U^{\mathrm{opt}}$ because it indicates the feasible UAV locations and always includes $U^{\mathrm{opt}}$. The $U^{\mathrm{opt}}$ will be determined to be on either $C_{a}$ or $C_{b}$ within $A_{C_{1}\cap C_{2}}$ (refer to Section 4.6 in [24]), especially near the upper point of intersection of $C_{a}$ and $C_{b}$ to minimize the interference between UAV and CU. Note that $A_{C_{1}\cap C_{2}}$ always exists due to the assumption at the beginning of Section 4 that the given transmit time allocation $\left(K_{no},K_{o}\right)$ guarantees the existence of feasible UAV locations.

We introduce two more cases $C_{1}^{=}$ and $C_{2}^{=}$, which represent that current UAV location satisfies the constraints (19a) and (19b) with an equality, respectively. Hence, it is clear that curve $C_{a}$ consists of $A_{C_{1}^{=}\cap C_{2}}$ and $A_{C_{1}^{=}\cap \bar{C_{2}}}$, whereas $C_{b}$ is composed of $A_{C_{1}\cap C_{2}^{=}}$ and $A_{\bar{C_{1}}\cap C_{2}^{=}}$. In addition, $A_{C_{1}\cap C_{2}}$ includes $A_{C_{1}^{=}\cap C_{2}}$ and $A_{C_{1}\cap C_{2}^{=}}$ (see Figure 3a).

The UD algorithm consists of three steps: [Step 1] for finding a feasible UAV location $U^{f}$ from an initial UAV location $U^{ini}$, [Step 2] for updating $U^{f}$ towards $U^{\mathrm{opt}}$, and [Step 3] for determining $U^{\mathrm{opt}}$ and terminating the algorithm. Figure 3b,d represent three steps respectively, and search directions along a line or a circle have the same properties as those in Figure 2.

#### 4.3.1. [Step 1] Finding $U^{f}$ from $U^{ini}$

To utilize Observations for search directions, it is essential to place an initial UAV at an arbitrary location. We suggest that the initial UAV location $U^{ini}=U^{U}$ be at the point of intersection of $C_{b}$ and line in Figure 2d with $\theta_{U,IU}\approx 90$ (e.g., ‘◆’ in Figure 3b). This point belongs to $A_{\bar{C_{1}}\cap C_{2}^{=}}$, hence Direction ➃ on Observation 7 can be applied to find $U^{f}=U^{U^{\prime }}$ by decreasing $d_{U,BS}$ along the line between BS and $U^{ini}$. Since this line *always* passes through $A_{C_{1}\cap C_{2}}$, the feasible UAV location $U^{f}$ can be found within $A_{C_{1}\cap C_{2}}$, specifically at the intersection of $A_{C_{1}^{=}\cap C_{2}}$ and the line (e.g., ‘●’ in Figure 3b) to minimize interference from UAV to CU. Therefore, $U^{f}=U^{U^{\prime }}=\left\{d_{U^{\prime },BS},\theta_{U,BS}\right\}$ can be obtained by directly calculating $d_{U^{\prime },BS}$ from (19a) as

(20) $$\begin{array}{c} d_{U^{\prime },BS}=X_{BS}^{-1}\left(F\left(\theta_{U,BS}\right)\;|K_{no}\right), \end{array}$$

where $X_{BS}^{-1}\left(\cdot \;|K_{no}\right)$ is the inverse function of $X_{BS}\left(\cdot |K_{no}\right)$ and $\theta_{U,BS}=\theta_{U^{\prime },BS}$ is the elevation angle between BS and $U^{ini}$.

Alternatively, $U^{ini}$ at the intersection of $C_{a}$ and line in Figure 2c with $\theta_{U,BS}\approx 90$ (e.g., ‘✚’ in Figure 3b) can be considered, and it is within $A_{C_{1}^{=}\cap \bar{C_{2}}}\subset A_{C_{1}\cap \bar{C_{2}}}$. Based on Direction ➃ on Observation 8, $U^{f}=U^{U^{\prime }}=\left\{d_{U^{\prime },IU},\theta_{U,IU}\right\}$ can be determined to be on $C_{b}$, where

(21) $$\begin{array}{c} d_{U^{\prime },IU}=X_{IU}^{-1}\left(F\left(\theta_{U,IU}\right)\;|K_{o}\right). \end{array}$$

It is obtained by taking the inverse function of $X_{IU}\left(\cdot |K_{o}\right)$, $X_{IU}^{-1}\left(\cdot \;|K_{o}\right)$, to (19b) with $\theta_{U,IU}=\theta_{U^{\prime },IU}$.

#### 4.3.2. [Step 2] Updating $U^{f}$

[Step 1] finds the feasible UAV location $U^{f}$ ($=U^{U}$ in this step) within $A_{C_{1}^{=}\cap C_{2}}$ on $C_{a}$, or $A_{C_{1}\cap C_{2}^{=}}$ on $C_{b}$. [Step 2] updates $U^{U}$ iteratively towards $U^{\mathrm{opt}}$ and near upper point of intersection of $C_{a}$ and $C_{b}$ as shown in Figure 3c. $A_{C_{1}^{=}\cap C_{2}}$ and $A_{C_{1}\cap C_{2}^{=}}$ belong to $A_{C_{1}\cap C_{2}}$, hence, Observation 6 can be applied. When $U^{U}$ is within $A_{C_{1}^{=}\cap C_{2}}$ on $C_{a}$ (e.g., ‘●’ in Figure 3c), $U^{U^{\prime }}=\left\{d_{U^{\prime },IU},\theta_{U,IU}\right\}$ will be on $C_{b}$ by increasing $d_{U,IU}$ along Direction ➀ in Figure 2d, according to (21). On the other hand, when $U^{U}$ is within $A_{C_{1}\cap C_{2}^{=}}$ on $C_{b}$ (e.g., ‘★’ in Figure 3c), $U^{U^{\prime }}=\left\{d_{U^{\prime },BS},\theta_{U,BS}\right\}$ will be obtained on $C_{a}$ by increasing $d_{U,BS}$ along Direction ➀ in Figure 2c based on (20). The newly obtained $U^{U^{\prime }}$ becomes $U^{U}$ for the next procedure to draw a line for search directions. These procedures iterate until $U^{U^{\prime }}$ locates on either $C_{a}$ or $C_{b}$ outside $A_{C_{1}\cap C_{2}}$, but near the upper intersection point of $C_{a}$ and $C_{b}$ (e.g., ‘✚’ in Figure 3c).

Note that, search directions along the line (i.e., Direction ➀ in Figure 2c,d) are selected on Observation 6 rather than those along the circle (i.e., Direction ➁ in Figure 2c,d). Since (19a) is not a function of $\theta_{U,IU}$, Direction ➁ in Figure 2d cannot find $U^{U^{\prime }}$ within $A_{C_{1}^{=}\cap C_{2}}$ (e.g., ‘●’ in Figure 3c) from $U^{U}$ within $A_{C_{1}\cap C_{2}^{=}}$ directly, but numerically by searching $\theta_{r,iu}$ as

(22) $$\begin{array}{cc} \theta_{U^{\prime },IU} & =\theta_{U,IU}+\min\Delta_{\theta }\\ \; & s.t.\;U^{U^{\prime }}\mathrm{satisfies}\;(19a)\;\mathrm{with}\;\mathrm{an}\;\mathrm{equality}, \end{array}$$

where $\Delta_{\theta }$ is an increment of $\theta_{U,IU}$ and $d_{U^{\prime },IU}=d_{U,IU}$. Similarly, Direction ➁ in Figure 2c requires numerical updating $\theta_{U,BS}$ to find $U^{U^{\prime }}$ within $A_{C_{1}\cap C_{2}^{=}}$ (e.g., ‘★’ in Figure 3c) from $U^{U}$ within $A_{C_{1}^{=}\cap C_{2}}$. These numerical updates increase computation time on Step 2, therefore, it is preferable to select Direction ➀ in Figure 2c and Direction ➀ in Figure 2d on Observation 6, thereby determining $U^{U^{\prime }}$ from (20) or (21) directly.

#### 4.3.3. [Step 3] Determining $U^{\mathrm{opt}}$

[Step 2] places $U^{U^{\prime }}$ at ‘✚’ in Figure 3c, which is outside $A_{C_{1}\cap C_{2}}$. [Step 3] puts it back at a point either on $C_{a}$ or $C_{b}$ within $A_{C_{1}\cap C_{2}}$, and then declares the optimal UAV location $U^{\mathrm{opt}}$.

Observations 7 and 8 can be utilized for this step because $U^{U^{\prime }}$ from [Step 2] (=$U^{U}$ in this step) is on either $C_{a}$ or $C_{b}$ outside $A_{C_{1}\cap C_{2}}$, specifically $A_{C_{1}^{=}\cap \bar{C_{2}}}$ or $A_{\bar{C_{1}}\cap C_{2}^{=}}$. When $U^{U}$ is within $A_{C_{1}^{=}\cap \bar{C_{2}}}$ (e.g., ‘✚’ in Figure 3d), Direction ➃ on Observation 8 can be applied using (21) to put $U^{U^{\prime }}$ on $C_{b}$, while Direction ➃ on Observation 7 can be utilized to place $U^{U^{\prime }}$ on $C_{a}$ using (20) when $U^{U}$ is within $A_{\bar{C_{1}}\cap C_{2}^{=}}$ (e.g., ‘◆’ in Figure 3d). If newly obtained $U^{U^{\prime }}$ is within $A_{C_{1}\cap C_{2}}$, more accurately $A_{C_{1}^{=}\cap C_{2}}$ or $A_{C_{1}\cap C_{2}^{=}}$, the UD algorithm declares that it is $U^{\mathrm{opt}}$, and terminates. If not, it repeats [Step 3] until $U^{U^{\prime }}$ is within $A_{C_{1}^{=}\cap C_{2}}$ or $A_{C_{1}\cap C_{2}^{=}}$. The details of UD algorithm are summarized in the Algorithm 1.
**Algorithm 1** UD algorithm.**Input**: ($K_{no}$, $K_{o}$). **Output**: Optimal UAV location $U^{\mathrm{opt}}$. **Step 1. Find $U^{f}$ from $U^{ini}$:**  1: initial UAV location is determined as $U^{ini}=\left\{d_{U,IU},\theta_{U,IU}\right\}$   $=\{X_{IU}^{-1}\left(F\left(\theta_{U,IU}\right)\;|K_{o}\right),\theta_{U,IU}\}$ with $\theta_{U,IU}\approx 90$.  2: update $U^{ini}$ following Direction ➃ in Figure 2c based on (20).   Then, $U^{f}$ within $A_{C_{1}^{=}\cap C_{2}}$ is obtained, and go to Step 2. **Step 2. Update $U^{f}$ towards $U^{\mathrm{opt}}$:**  3: **if **
$U^{f}$ is within $A_{C_{1}^{=}\cap C_{2}}$ **then**  4: find $U^{U^{\prime }}$ following Direction ➀ in Figure 2d based on (21).  5: **else if**
$U^{f}$ is within $A_{C_{1}\cap C_{2}^{=}}$ **then**  6: find $U^{U^{\prime }}$ following Direction ➀ in Figure 2c based on (20).  7: **end if**  8:**if**
$U^{U^{\prime }}$ is within $A_{C_{1}^{=}\cap \bar{C_{2}}}$ **then**  9: $U^{U}\leftarrow U^{U^{\prime }}$, and go to Step 3.  10: **else**  11: $U^{f}\leftarrow U^{U^{\prime }}$, and go to line 3.  12: **end if** **Step 3. Determine $U^{\mathrm{opt}}$:**  13: **if**
$U^{U}$ is within $A_{C_{1}^{=}\cap \bar{C_{2}}}$ **then**  14: find $U^{U^{\prime }}$ following Direction ➃ in Figure 2d based on (21).  15: **else if**
$U^{U}$ is within $A_{{\bar{C}}_{1}\cap C_{2}^{=}}$ **then**  16: find $U^{U^{\prime }}$ following Direction ➃ in Figure 2c based on (20).  17: **end if**  18: **if**
$U^{U^{\prime }}$ is within $A_{C_{1}^{=}\cap C_{2}}$ or $A_{C_{1}\cap C_{2}^{=}}$ **then**  19: $U^{opt}\leftarrow U^{U^{\prime }}$, and terminates the algorithm.  20: **else**  21: go to line 13.  22: **end if**

## 5. Optimal Number of Transmit Time Slots

To maximize the multi-objective function of **JUDTAP** in (12), the overall number of time slots *n* that guarantees a reliable relay transmission to IU should be minimized before UD algorithm is performed. Hence, in this section, we propose the time slot determination (TSD) algorithm to determine an optimal pair of time slots ($K_{no}^{opt}$, $K_{o}^{opt}$), equivalently the minimum (optimal) number of overall time slots of $n^{opt}=K_{no}^{opt}+K_{o}^{opt}$.

### 5.1. Existence of Feasible UAV Locations

In Section 4, UD algorithm is proposed to find $U^{\mathrm{opt}}$ with the assumption that the given time allocation ($K_{no}$, $K_{o}$) guarantees the existence of feasible UAV locations, equivalently $A_{C_{1}\cap C_{2}}$, that satisfies both constraint (19a) and (19b). However, when $K_{no}$ and $K_{o}$ are not enough for reliable relay transmissions, $A_{C_{1}\cap C_{2}}$ in Figure 3a does not exist for $U^{\mathrm{opt}}$. Therefore, it is critical to select a proper pair of ($K_{no}$, $K_{o}$), but we consider the minimum $K_{no}$ and $K_{o}$ as an optimum in the resource-efficiency aspect. In addition, scanning all UAV operation range (i.e., inside dashed rectangular in Figure 2) to check the existence of $A_{C_{1}\cap C_{2}}$ for each possible ($K_{no}$, $K_{o}$) is impractical. Therefore, a time-efficient determination for the existence of $A_{C_{1}\cap C_{2}}$ should be considered. This can be realized by utilizing [Step 1] of UD algorithm. If $U^{f}$ within $A_{C_{1}^{=}\cap C_{2}}$ can be found from $U^{ini}$, $A_{C_{1}\cap C_{2}}$ exists for ($K_{no}$, $K_{o}$) to guarantee a reliable relay transmission. On the other hand, if [Step 1] cannot find $U^{f}$ within $A_{C_{1}^{=}\cap C_{2}}$, ($K_{no}$, $K_{o}$) is not enough to satisfy both constraints on relay transmission (12a), thereby resulting in absence of $A_{C_{1}\cap C_{2}}$. Hence, more time slots should be allocated for a reliable relay transmission.

### 5.2. Time Slot Determination (TSD) Algorithm

In this section, we propose a novel time slot determination (TSD) algorithm to derive the minimum number of overall time slots $n^{opt}$ along with ($K_{no}^{opt}$, $K_{o}^{opt}$) for a reliable relay transmission. First, it defines $l_{min}$ and $l_{max}$ with the assumption of $K_{no}=K_{o}$, and utilizes them to reduce the search range for $K_{no}^{opt}$ and $K_{o}^{opt}$. $l_{min}$ is the minimum number of time slots where $(K_{no}$, $K_{o})=(l_{min}$,$l_{min})$ could provide $A_{C_{1}\cap C_{2}}$, but not necessarily guarantee it, whereas $l_{max}$ is the number of time slots that does guarantee $A_{C_{1}\cap C_{2}}$ for $(K_{no}$, $K_{o})=(l_{max}$,$l_{max})$. $l_{min}$ is determined by the upper bounds of $d_{U,BS}$ and $d_{U,IU}$, denoted as $d_{U,BS}^{max}\left(K_{no}\right)$ and $d_{U,IU}^{max}\left(K_{o}\right)$, while $l_{max}$ is obtained based on $l_{min}$ and utilized to find ($K_{no}^{opt}$, $K_{o}^{opt}$) and $n^{opt}$.

#### 5.2.1. Determination of $l_{min}$

The $l_{min}$ aims at restricting search range, thereby reducing a computation time for ($K_{no}^{opt}$, $K_{o}^{opt}$). It does not require guaranteeing $A_{C_{1}\cap C_{2}}$ necessarily, but provides a lower bound for $l_{max}$. It can be derived from maximum distances of $d_{U,BS}$ and $d_{U,IU}$, $d_{U,BS}^{max}\left(K_{no}\right)$ and $d_{U,IU}^{max}\left(K_{o}\right)$, for each $K_{no}$ and $K_{o}$. From (15) and (16), $d_{U,BS}\leq X_{BS}^{-1}\left(F\left(\theta_{U,BS}\right),K_{no}\right)$ and $d_{U,IU}\leq X_{IU}^{-1}\left(F\left(\theta_{U,IU}\right),K_{o}\right)$ can be obtained, and the right terms of both inequalities are increasing functions of $F(\theta_{U,BS})$ and $F(\theta_{U,IU})$ respectively. Hence, $d_{U,BS}^{max}\left(K_{no}\right)$ and $d_{U,IU}^{max}\left(K_{o}\right)$ can be defined as

(23) $$\begin{array}{cc} d_{U,BS}^{max}\left(K_{no}\right)\; & ≜\;X_{BS}^{-1}{\left(F\left(\theta_{U,BS}\right),K_{no}\right)}_{|F(\theta_{U,BS})=1}=X_{BS}^{-1}\left(\;1,K_{no}\right), \end{array}$$

(24) $$\begin{array}{cc} d_{U,IU}^{max}\left(K_{o}\right)\; & ≜\;X_{IU}^{-1}{\left(F\left(\theta_{U,IU}\right),K_{o}\right)}_{|F(\theta_{U,IU})=1}=X_{IU}^{-1}\left(\;1,K_{o}\right), \end{array}$$

where $F(\theta_{U,i})=1,i\in \left\{BS,IU\right\}$ because $\max F(\theta_{U,I})=1$. Note that $d_{U,BS}^{max}\left(K_{no}\right)$ and $d_{U,IU}^{max}\left(K_{o}\right)$ are increasing functions of $K_{no}$ and $K_{o}$, respectively.

Using (23) and (24), $l_{min}$ is defined as

(25) $$\begin{array}{c} l_{\min}=\underset{z\in Z_{>0}}{\arg\min}\;d_{U,BS}^{max}\left(z\right)+d_{U,IU}^{max}\left(z\right)\geq d_{BS,IU}, \end{array}$$

where *z* is a positive integer indicating $z=K_{no}=K_{o}$, and $d_{BS,IU}$ is a distance between BS and IU. The $l_{\min}$ given by (25) makes two circles drawn at ground node *i* with radius $d_{U,i}^{max}\left(l_{\min}\right)$, i∈{BS,IU} (i.e., blue and dashed green circles in Figure 2a) overlap each other. As aforementioned, the feasible UAV locations could exist within an overlapped region by two circles, but not guaranteed because the derivation of $l_{min}$ starts with the assumption of $F(\theta_{U,i})=1,i\in \left\{BS,IU\right\}$ in (23) and (24).

#### 5.2.2. Determination of $l_{max}$

If a feasible UAV location exists for ($K_{no},K_{o})=(l_{\min},l_{\min}$), $l_{max}$ can be determined as $l_{max}≜l_{min}$ by the definition. Otherwise, $l_{max}$ should be greater than $l_{min}$ such that a feasible UAV location exists for $\left(K_{no},K_{o}\right)=\left(l_{\max},l_{\max}\right)$. In addition, $K_{no}$ and $K_{o}$, both less than $l_{min}$, do not need to be considered because it can not provide $A_{C_{1}\cap C_{2}}$. Hence, $l_{max}$ can be obtained as

(26) $$\begin{array}{cccc} l_{\max} & = & \; & \;\;\underset{l_{\min}+z}{\arg\min}\left(l_{\min}+z,l_{\min}+z\right),\\ s.t & \; & & z\in Z_{\geq 0}and\\ \; & \; & & A_{C_{1}\cap C_{2}}\;\mathrm{exists}\;\mathrm{for}\;\left(K_{no},K_{o}\right)=\left(l_{\min}\;+\;z,l_{\min}\;+\;z\right), \end{array}$$

where *z* is a non-negative integer value. If a feasible UAV location exists for $\left(K_{no},K_{o}\right)=\left(l_{\min},l_{\min}\right)$, z=0, and otherwise, z>0. Note that it is obvious that $n^{opt}\leq 2l_{\max}$ because a feasible UAV location always exists for $\left(K_{no},K_{o}\right)=\left(l_{\max},l_{\max}\right)$.

#### 5.2.3. Determination of ($K_{no}^{opt},K_{o}^{opt}$)

Based on $l_{max}$, the initial number of overall time slots is defined as $n_{ini}=2l_{max}$. The TSD algorithm targets to the minimum number of overall time slots, so $K_{no}$ and $K_{o}$ can be different even though $n_{ini}$ is derived from the assumption of $K_{no}=K_{o}$. In addition, for each number of overall time slot *n*, it is preferable to maximize $K_{no}$ to achieve maximum $R_{CU}$, because the SINR at CU in NOTP (i.e., (2)) is larger than that in OTP (i.e., (4)). Therefore, $n^{opt}$ will be determined by any pair of ($K_{no},K_{o}$), which leads to the minimum $K_{no}+K_{o}$. However, when $n^{opt}$ is given, ($K_{no}^{opt},K_{o}^{opt})$ is preferred to be ($K_{no},K_{o}$) with $K_{no}\geq K_{o}$ if exists.

**Figure 4:**
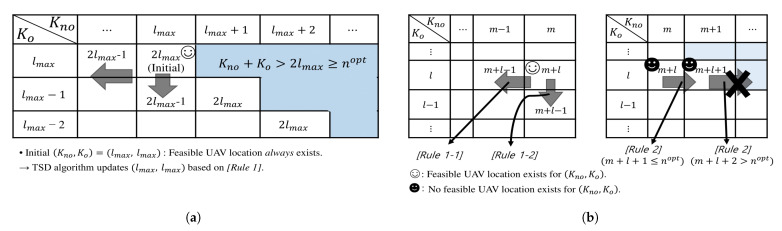
Part 2 on the time slot determination (TSD) algorithm. (**a**) Updating initial $\left(K_{no},K_{o}\right)$ = $\left(l_{max},l_{max}\right)$. (**b**) Updating rules for $\left(K_{no},K_{o}\right)$.

Figure 4 represents a way to update $K_{no}$ and $K_{o}$ towards $K_{no}^{opt}$ and $K_{o}^{opt}$. The row and column of the table indicate values of $K_{o}$ and $K_{no}$. $K_{o}$ is upper bounded by $l_{max}$ because $\left(K_{no},K_{o}\right)=\left(l_{\max}-z,l_{\max}+z\right)$, $z\in Z_{>0}$ for $n_{ini}=2l_{\max}$ cannot achieve $K_{no}\geq K_{o}$. Each element of table denotes the sum of row and column values (i.e., the number of overall time slots), and pairs of $K_{no}$ and $K_{o}$ resulting in $K_{no}+K_{o}>2l_{max}$ are out of interest (i.e., upper triangle in Figure 4). To find ($K_{no}^{opt},K_{o}^{opt}$), an initial time allocation is set to $\left(K_{no},K_{o}\right)=\left(l_{max},l_{max}\right)$, $n^{opt}=n_{ini}$, and two rules for updating $\left(K_{no},K_{o}\right)$ are defined as follows:

[Rule 1]

If a feasible UAV location exists for $\left(K_{no},K_{o}\right)=\left(m,l\right)$, update $n^{opt}$ as m+l and check the existence of feasible UAV location for $\left(K_{no},K_{o}\right)=\left(m,l-1\right)$ and $\left(K_{no},K_{o}\right)=\left(m-1,l\right)$, obtained by downward and leftward movements in Figure 4 to decrease $K_{o}$ and $K_{no}$, respectively.

[Rule 1-1] If a feasible UAV location exists *only* for $\left(K_{no},K_{o}\right)=\left(m-1,l\right)$, update $n^{opt}$ as m+l−1 and repeat [Rule 1] at $\left(K_{no},K_{o}\right)=\left(m-1,l\right)$.

[Rule 1-2] If a feasible UAV location exists for $\left(K_{no},K_{o}\right)=\left(m,l-1\right)$, update $n^{opt}$ as m+l−1 and repeat [Rule 1] at $\left(K_{no},K_{o}\right)=\left(m,l-1\right)$.

[Rule 2]

If no feasible UAV location exists for $\left(K_{no},K_{o}\right)=\left(m,l\right)$ and $m+l+1\leq n^{opt}$, move right in Figure 4 to increase $K_{no}$ and check the existence of feasible UAV location for $\left(K_{no},K_{o}\right)=\left(m+1,l\right)$.

[Rule 1] aims at checking the availability of smaller $n^{opt}$, while [Rule 2] is to investigate the existence of $\left(K_{no},K_{o}\right)$ with $K_{no}>K_{o}$ for the candidate $n^{opt}$. When feasible UAV locations exist for both $\left(K_{no},K_{o}\right)=\left(m-1,l\right)$ and $\left(K_{no},K_{o}\right)=\left(m,l-1\right)$ in [Rule 1], $\left(K_{no},K_{o}\right)=\left(m,l-1\right)$ in [Rule 1-2] is selected to maximize $K_{no}$ for $n^{opt}=m+l-1$. TSD algorithm terminates when $\left(K_{no},K_{o}\right)=\left(m+1,l\right)$ in [Rule 2] results in $m+l+1>n^{opt}$. The last updated $n^{opt}$ is minimum (optimal) number of overall time slots, and $\left(K_{no},K_{o}\right)$, which leads to $n^{opt}$, becomes $\left(K_{no}^{opt},K_{o}^{opt}\right)$.

#### 5.2.4. TSD Algorithm

Details of the TSD algorithm are summarized in Algorithm 2. Part 1 determines $l_{min}$ and $l_{max}$ from (25) and (26), respectively. Part 2 derives $n^{opt}$ and $\left(K_{no}^{opt},K_{o}^{opt}\right)$ from ($K_{no},K_{o}$) = ($l_{max},l_{max}$) by the updating rules for ($K_{no},K_{o}$). Note that only a few iterations are required on Part 2 of the TSD algorithm. From (26), it is clear that $\left(K_{no},K_{o}\right)=\left(l_{\max}-1,l_{\max}-1\right)$ with $n=2l_{\max}-2$ does not provide a feasible UAV location. This results from the assumption of $K_{no}=K_{o}$, hence, $n=2l_{\max}-2$ could give a feasible UAV location when $K_{no}$ is different from $K_{o}$. However, there is little chance for such a case to obtain a valid $\left(K_{no},K_{o}\right)$ with $n=2l_{\max}-2$ because $K_{no}$ or $K_{o}$ may be too small to set a reliable relay link between BS and UAV or between UAV and IU. In other words, a leftward or a downward movement in Figure 4 may be enough once or twice to reach $n^{opt}$, and so may a rightward movement to maximize $K_{no}$ for the same reason. Therefore, the TSD algorithm reduces a search time dramatically compared to exhaustive algorithm or others, hence delivers $K^{\mathrm{opt}}=\left(K_{no}^{opt},K_{o}^{opt}\right)$ quickly.
**Algorithm 2** TSD algorithm.**Part 1. Calculate of $l_{\min}$ and $l_{\max}$**.  1: Determine $l_{\min}$ using (25).  2: Determine $l_{\max}$ using (26). **Part 2: Find ($K_{no}^{opt},K_{o}^{opt})$**. **Input**: $\left(l_{\max},l_{\max}\right)$. **Output**: ($K_{no}^{opt},K_{o}^{opt}),n^{opt}$.  Initialization: $\left(K_{no},K_{o}\right)$ ← $\left(l_{\max},l_{\max}\right)$, $n^{opt}\leftarrow 2l_{\max}$.  3: **if** feasible UAV location exists for $\left(K_{no},K_{o}\right)$ **then**  4:**if** feasible UAV location exists for $\left(K_{no},K_{o}-1\right)$ **then**  5:  ($K_{no}^{opt},K_{o}^{opt})\leftarrow \left(K_{no},K_{o}-1\right)$, $n^{opt}\leftarrow K_{no}+K_{o}-1$.  6:  $\left(K_{no},K_{o}\right)\leftarrow \left(K_{no},K_{o}-1\right)$, and go to line 3.  7: **else if** feasible UAV location exists for $\left(K_{no}-1,K_{o}\right)$ **then**  8:  ($K_{no}^{opt},K_{o}^{opt})\leftarrow \left(K_{no}-1,K_{o}\right)$, $n^{opt}\leftarrow K_{no}+K_{o}-1$.  9:  $\left(K_{no},K_{o}\right)\leftarrow \left(K_{no}-1,K_{o}\right)$, and go to line 3.  10: **end if**  11: **else**  12: **if**  $K_{no}+K_{o}+1\leq n^{opt}$
**then**  13:  $\left(K_{no},K_{o}\right)\leftarrow \left(K_{no}+1,K_{o}\right)$, and go to line 3.  14: **else**  15:  Terminate the algorithm.  16: **end if**  17: **end if**

## 6. UAV Deployment and Time Allocation Algorithm

The TSD and UD algorithms are presented to determine $K^{\mathrm{opt}}$=$\left\{K_{no}^{opt},K_{o}^{opt}\right\}$ and to decide $U^{\mathrm{opt}}$ for $K^{\mathrm{opt}}$, respectively. The **JUDTAP** can be solved by UAV deployment and transmit time allocation (UDTA) algorithm, which consists of TSD and UD algorithms and runs them in a sequential manner. Details of UDTA algorithm are summarized in Algorithm 3. As mentioned in Section 4 and Section 5, TSD and UD algorithms reduce search range for $K^{\mathrm{opt}}$ and search area for $U^{\mathrm{opt}}$ respectively, thereby requiring much less computation time over exhaustive search algorithm. In the following subsection, a computational complexity is analyzed with respect to the total number of computations, considered for searching and determining $K^{\mathrm{opt}}$ and $U^{\mathrm{opt}}$.
**Algorithm 3** UDTA algorithm.**Input**: $D_{req}$, $x_{BS}$, $x_{CU}$, and $x_{IU}$. **Output**: $K^{\mathrm{opt}}$, $U^{\mathrm{opt}}$. **Part 1: Optimal number of time slots**.  1: Find ${ł}_{\min}$ and ${ł}_{\max}$ from Part 1 on TSD algorithm.  2: Find $K^{\mathrm{opt}}=$($K_{no}^{opt},K_{o}^{opt})$ from Part 2 on TSD algorithm. **Part 2: Optimal UAV location**.  3: Based on ($K_{no}^{opt},K_{o}^{opt})$, find $U^{\mathrm{opt}}$ using UD algorithm.


### Complexity Analysis

To determine the number of UAV locations for an exhaustive search, we consider that a grid is superimposed over the operation range (i.e., dashed rectangular in Figure 2a) with lines separated by $\Delta_{d}$ [m]. As a result, the number of UAV locations to be considered increases as $\Delta_{d}$ decreases. Total computations for an exhaustive search is derived as $O\left(\Delta_{d}\right)≜{\left\lceil \frac{S}{{\Delta_{d}}^{2}}\right\rceil }^{\frac{n^{opt}\left(n^{opt}-1\right)}{2}}$. $\left\lceil \frac{S}{{\Delta_{d}}^{2}}\right\rceil$ is the number of UAV locations within the operation range of *S* [$m^{2}$], where $\left\lceil \cdot \right\rceil$ is rounding up to the nearest integer. $\frac{n^{opt}\left(n^{opt}-1\right)}{2}$ is the number of combinations for $\left(K_{no},K_{o}\right)$ to find $\left(K_{no}^{opt},K_{o}^{opt}\right)$ from initial $\left(K_{no},K_{o}\right)$ = (1,1). Hence, $O(\Delta_{d})$ indicates that $\left\lceil \frac{S}{{\Delta_{d}}^{2}}\right\rceil$ locations for their feasibility need to be considered for each time allocation $\left(K_{no},K_{o}\right)$. On the other hand, the UDTA algorithm requires at most $l_{min}+2^{\left|l_{max}-l_{min}\right|}+2^{2\left|l_{max}-K_{o}^{opt}\right|+\left|l_{max}-K_{no}^{opt}\right|}+10$ number of computations to find $K^{\mathrm{opt}}$ and $U^{\mathrm{opt}}$. On TSD algorithm, $l_{min}$ computations are required in (25) to determine $l_{min}$ from $\left(K_{no},K_{o}\right)=\left(1,1\right)$. To determine $l_{max}$, $2^{\left|l_{max}-l_{min}\right|}$ computations are required in (26) from $l_{min}$, where only two UAV locations (i.e., initial and first feasible locations in [Step 1] on UD algorithm) are considered for each time allocation $\left(K_{no},K_{o}\right)$. Similarly, there are at most 2$\left|l_{max}-K_{o}^{opt}\right|+\left|l_{max}-K_{no}^{opt}\right|$ combinations for $\left(K_{no},K_{o}\right)$ to find $\left(K_{no}^{opt},K_{o}^{opt}\right)$ from $\left({ł}_{max},{ł}_{max}\right)$, hence resulting in $2^{2\left|l_{max}-K_{o}^{opt}\right|+\left|l_{max}-K_{no}^{opt}\right|}$ computations. In the UD algorithm, the total number of computations in (20) or (21) is less than 10, which is derived from simulations and reasonable due to dramatically reduced search area within $A_{C_{1}\cap C_{2}}$ by the proposed algorithm. As a result, the UDTA algorithm requires much fewer UAV locations and $\left(K_{no},K_{o}\right)$ combinations to be considered for $K^{\mathrm{opt}}$ and $U^{\mathrm{opt}}$ over exhaustive search algorithm, thereby reducing computational time and effort significantly.

## 7. Numerical Results

In this section, we compare the optimal UAV location and transmit time allocations by proposed algorithms with those from an exhaustive search, and demonstrate that the UDTA algorithm achieves optimality while significantly reducing computational complexity. For simulations, we assume that $P_{BS}^{max}$ = 30 [dBm], $P_{U}^{max}$ = 25 [dBm], $\beta_{A}=3$, $\beta_{G}=2$, and ς = 20 [dB] [25,26,27,28]. An urban environment is assumed with B=0.136 and C=11.95 [8]. $\lambda ≜\frac{d_{BS,IU}}{d_{BS,CU}}$ is a relative location of IU with respect to CU. In order to evaluate the optimality of proposed algorithm, the throughput gap (%) is defined by $R_{CU}$ difference from exhaustive search of $\Delta_{d}=1$ because $\Delta_{d}=1$ is sufficiently small to find the global optimal UAV location for exhaustive search.

**Figure 5:**
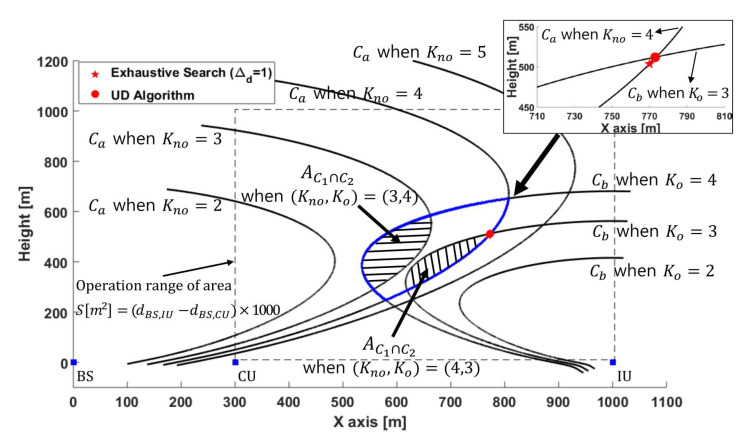
Feasible combinations of $\left(K_{no},K_{o}\right)$ and comparison of $U^{\mathrm{opt}}$ from different algorithms.

Figure 5 represents feasible combinations of $\left(K_{no},K_{o}\right)$ and formation of $A_{C_{1}\cap C_{2}}$, and compares $U^{\mathrm{opt}}$ by UD algorithm with that by exhaustive search of $\Delta_{d}=1$, where $x_{BS}=0$, $x_{CU}=300$ [m], $x_{IU}=1000$ [m] and $D_{req}=3$ [bit/Hz]. When $\left(K_{no},K_{o}\right)=\left(4,4\right)$, $A_{C_{1}\cap C_{2}}$ starts to appear, but TSD algorithm concludes $\left(K_{no}^{opt},K_{o}^{opt}\right)=\left(4,3\right)$ resulting in $n^{opt}=7$, even though $\left(K_{no},K_{o}\right)=\left(3,4\right)$ also provides $A_{C_{1}\cap C_{2}}$. This is because $\left(K_{no}^{opt},K_{o}^{opt}\right)=\left(4,3\right)$ maximizes $K_{no}^{opt}$ for given $n^{opt}$. TSD algorithm reduces the feasible UAV locations $A_{C_{1}\cap C_{2}}$ significantly, and UD algorithm successfully determines $U^{\mathrm{opt}}$ close to that from the exhaustive search of $\Delta_{d}=1$.

**Figure 6:**
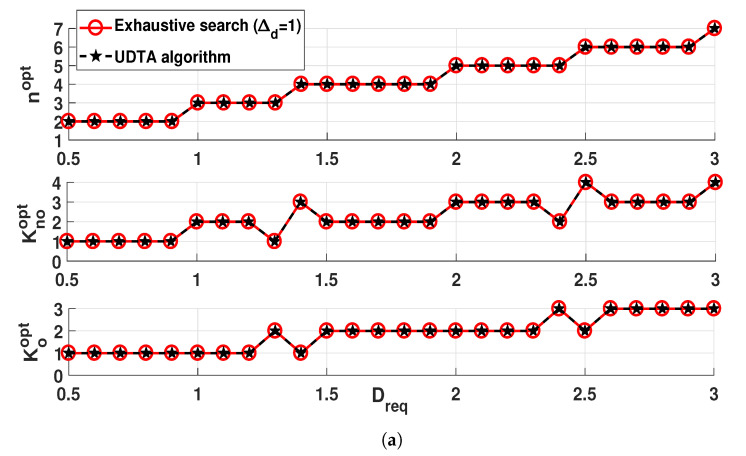

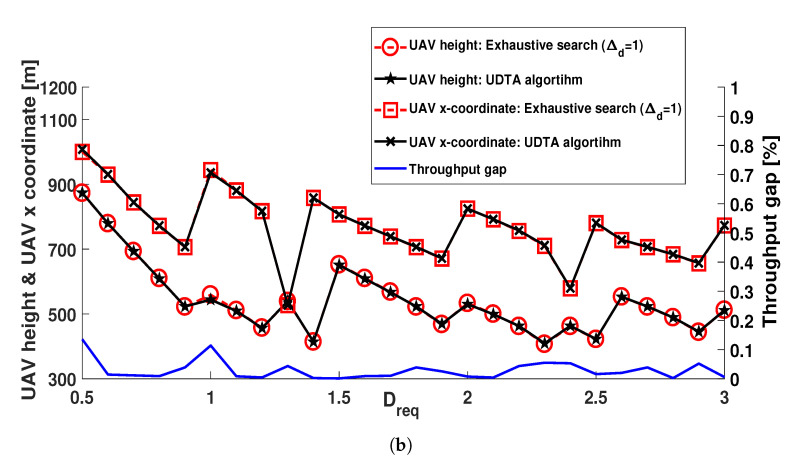
Performance comparison of different algorithms and their throughput gap with respect to $D_{req}$. (**a**) The number of transmit time slots. (**b**) UAV location and throughput gap.

Figure 6 represents $\left(K_{no}^{opt},K_{o}^{opt}\right)$, $n^{opt}$, UAV location, and throughput gap with respect to $D_{req}$ for same $x_{v}$, v∈{BS,CU,IU} in Figure 5. The UDTA algorithm finds $\left(K_{no}^{opt},K_{o}^{opt}\right)$ and UAV location close to optimum with negligible throughput gap. $n^{opt}$ increases as $D_{req}$ increases to set a reliable relay connection. For a given $n^{opt}$, UAV should be placed lower and close to CU as $D_{req}$ increases. Even though this UAV movement increases the interference to CU, it is necessary for reliable relay transmission in BS-to-UAV link. For example, Figure 6a represents that $n^{opt}=2$ is required for $0.5\leq D_{req}\leq 0.9$. When $D_{req}=0.5$, UAV can be placed very high and remote from CU. As $D_{req}$ increases, however, UAV moves towards BS to set a reliable BS-to-UAV link. Lastly, the throughput gap between the proposed algorithm and exhaustive search is less than 0.1 (%) for entire range of $D_{req}$, hence, it demonstrates that UDTA algorithm successfully determines $K^{\mathrm{opt}}$ and $U^{\mathrm{opt}}$ with negligible throughput gap from the exhaustive search of $\Delta_{d}=1$.

URN with multiple transmit time slots ($n^{opt}>$2) on each relay transmission link can reduce redundant usages of transmit time slots. For example, URN consisting of two time slots for a single relay transmission (i.e., single transmit time slot allocation to each relay transmission link) requires at least three repetitions of relay transmissions to provide $D_{req}=1.9$ at IU since $D_{req}=0.9$ is a maximum delivered data to IU by URN with $n^{opt}=2$, as shown in Figure 6a. Therefore, six time slots are required for URN that utilizes two transmit time slots for a single relay transmission, while URN that allocates multiple transmit time slots on each relay transmission link only requires four time slots for $D_{req}=1.9$. Hence, multiple transmit time slots should be adopted in URN to efficiently utilize the transmit time slots.

**Figure 7:**
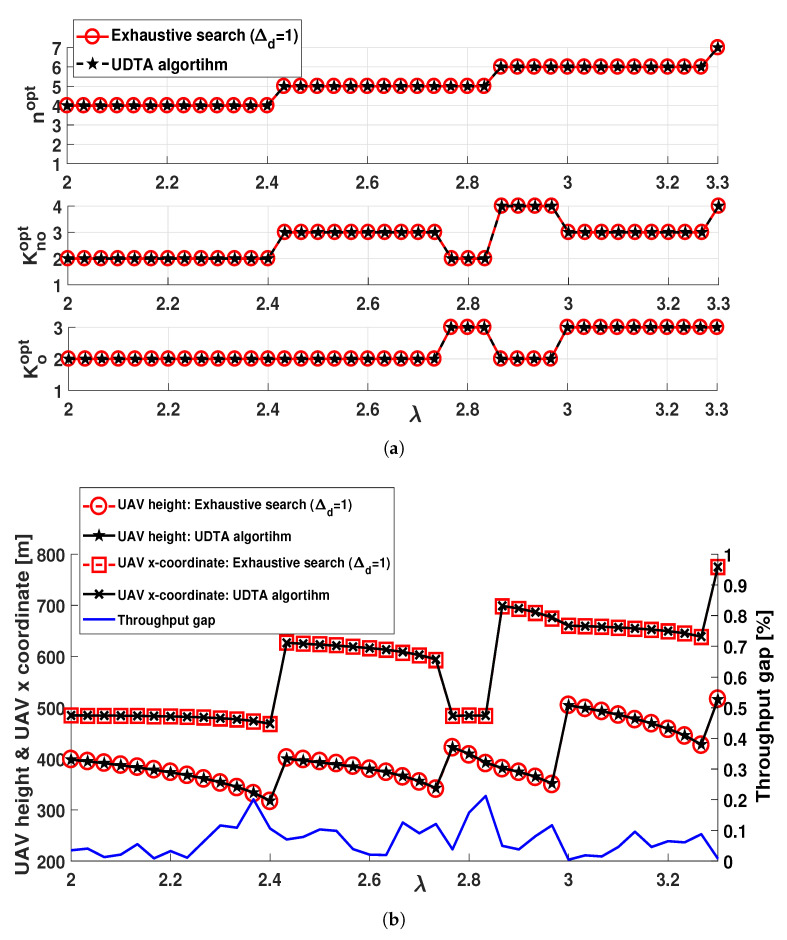
Performance comparison of different algorithms and their throughput gap with respect to λ. (**a**) The number of transmit time slots. (**b**) UAV location and throughput gap.

Figure 7 represents $\left(K_{no}^{opt},K_{o}^{opt}\right)$, $n^{opt}$, UAV location, and throughput gap with respect to λ for $x_{CU}=300$ [m]. As λ increases, IU moves away from BS, hence, larger $n^{opt}$ is required in URN for a reliable relay transmission. In addition, UAV height should be increased for large $\theta_{U,v}$ to set a strong LOS connection between UAV and ground nodes *v*. In order to guarantee the minimum number of transmit time slots, $K_{no}^{opt}$ can be smaller than $K_{o}^{opt}$ as explained in Section 5.2.3. For example, when 2.43≤λ≤2.83, $n^{opt}$ is equal to 5 with $\left(K_{no}^{opt},K_{o}^{opt}\right)=\left(3,2\right)$ or (2,3). Especially, $\left(K_{no}^{opt},K_{o}^{opt}\right)=\left(2,3\right)$ is selected when 2.76≤λ≤2.83 to achieve the minimum number of overall time slots, however, it requires UAV to move towards BS for reliable BS-to-UAV link due to smaller $K_{no}^{opt}$. Similar to Figure 6, UDTA algorithm achieves negligible throughput gap, less than 0.2 (%), over the exhaustive search of $\Delta_{d}=1$.

**Figure 8:**
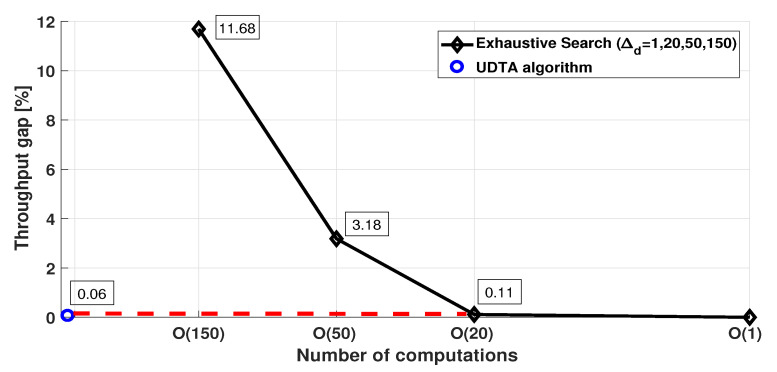
Throughput gap of exhaustive searches of different $\Delta_{d}$.

Figure 8 shows the throughput gap of exhaustive searches with respect to the number of computations. It is obvious that the throughput gap increases as $\Delta_{d}$ increases, due to the reduction on the number of UAV locations considered for searching $K^{\mathrm{opt}}$ and $U^{\mathrm{opt}}$, compared with $\Delta_{d}=1$. As aforementioned, the UDTA algorithm significantly reduces computational time to find $K^{\mathrm{opt}}$ and $U^{\mathrm{opt}}$ due to the time-efficient determination of $\left(K_{no}^{opt},K_{o}^{opt}\right)$ based on $l_{min}$ and $l_{max}$, and small $A_{C_{1}\cap C_{2}}$ derived from $\left(K_{no}^{opt},K_{o}^{opt}\right)$. Therefore, it provides optimal solution for **JUDTAP**, and achieves much less computations of $l_{min}+2^{\left|l_{max}-l_{min}\right|}+2^{2\left|l_{max}-K_{o}^{opt}\right|+\left|l_{max}-K_{no}^{opt}\right|}+10\ll O\left(1\right)$ with negligible throughput gap over the exhaustive search of even $\Delta_{d}\leq 1$.

## 8. Conclusions

In this paper, we have investigated URN with multiple transmit time slots, and proposed algorithms to maximize the throughput of UE in a cell while guaranteeing a reliable transmission to UE in its extended service area. The formulated multi-objective joint UAV deployment and transmit time allocation optimization problem (**JUDTAP**) is solved by TSD and UD algorithms to determine the optimal number of overall transmit time slots $K^{\mathrm{opt}}$ and optimal UAV location $U^{\mathrm{opt}}$ in a sequential manner. Simulation results demonstrate that $K^{\mathrm{opt}}$ and $U^{\mathrm{opt}}$ are critical to URN for a reliable relay transmission. $K^{\mathrm{opt}}$ and $U^{\mathrm{opt}}$ by the proposed algorithm match well those from exhaustive search, but with significantly reduced computation complexity to determine them over the exhaustive search. In addition, URN allocating multiple transmit time slots on relay transmission links is better than that utilizing two transmit time slots for a single relay transmission in terms of resource efficiency.

## Data Availability

Data is contained within the article.
